# Patient satisfaction and oral health-related quality of life 10 years after implant placement

**DOI:** 10.1186/s12903-020-01381-3

**Published:** 2021-01-14

**Authors:** Yan Wang, Daniel Bäumer, Ann-Kathrin Ozga, Gerd Körner, Amelie Bäumer

**Affiliations:** 1 Private Practice, Munich, Germany; 2 Institute of Medical Biometry and Epidemiology, University Medical Center Hamburg-Eppendorf, 20246 Hamburg, Germany; 3 Institute of Medical Biometry and Informatics, University of Heidelberg, 69120 Heidelberg, Germany; 4 Private Practice, Niedernstrasse 16, 33602 Bielefeld, Germany; 5 Section of Periodontology, Department of Conservative Dentistry, Clinic for Oral, Dental and Maxillofacial Diseases, University Hospital Heidelberg, Im Neuenheimer Feld 400, 69120 Heidelberg, Germany

**Keywords:** Implants, Patient satisfaction, Aesthetics, Quality of life, OHIP, PIDAQ

## Abstract

**Background:**

Implant survival and implant success (freedom of biologic complications) are important factors in assessing the success of implant therapy. However, these factors are not the only determinants. Patients’ satisfaction also plays a very important role in daily practice. Therefore, the aim of this study was to assess patients’ satisfaction regarding function (phonetics, chewing comfort, stability, cleanability) and aesthetics in patients treated with XiVE and Frialite implants in a private periodontal practice ten years after implant placement. Furthermore, oral health-related quality of life (OHRQoL) was evaluated.

**Methods:**

Patient-reported outcome measures (PROMs) regarding overall satisfaction, phonetics, chewing comfort, stability, cleanability, and aesthetics were examined on a Visual Analog Scale (VAS) 10 years ± 6 months after implant placement in a cross-sectional survey. OHRQoL and psychological impact were assessed via the *Oral Health Impact Profile* (OHIP) and *Psychosocial Impact of Dental Aesthetics Questionnaire* (PIDAQ). Potential influence of patient-related factors (age, gender, smoking, peri-implantitis, implant position, type of restoration) on VAS, OHRQoL and PIDAQ were investigated using regression analyses.

**Results:**

High satisfaction with implant-supported restorations was seen in all 95 patients ten years after implant placement. Mean VAS-score for general satisfaction with implant-supported restoration was 93.0% (SD ± 9.4, median: 96.3%, range 50.0–100%). Mean OHIP score was 11.3 (SD ± 10.8, median: 9.0, range 0–45), mean PIDAQ score 20.5 (SD ± 11.37, median: 17.0, range 0–52). A slight tendency that presence of a moderate/severe peri-implantitis lowers satisfaction could be detected (overall satisfaction: ordinal, *p* = 0.012, VAS, *p* = 0.026). Also, the factors age, implant position and type of restoration might have an impact on patient’s satisfaction.

**Conclusions:**

Patients restored with mostly fixed implant-supported restorations showed a very high patient satisfaction regarding function and aesthetics 10-year after implant placement. The presence of a moderate/severe peri-implantitis showed a slight tendency for influencing patient satisfaction. Due to the cross-sectional design results have to be interpreted with care.

## Background

Implant-supported restorations have become standard for the therapy of lost or missing teeth. Survival rates of implants are high [1, 2]. However, the time an implant remains in situ is not the only factor determining the success of implant therapy. Much more relevant for implant success is, whether the hard and soft tissues around implants are free of inflammation. Other important aspects are functional and aesthetic outcomes of the implant-supported restorations. Altogether, these criteria are essential for the aspired improvement in life quality. Patients experience the improvement of life quality individually. This phenomenon is referred to as patients satisfaction [3–5].

Health is one of the important dimensions regarding quality of life (c). Even though oral diseases with local symptoms, such as pain or tooth loss, are generally not life threatening, they can greatly affect the health-related quality of life (called oral health-related quality of life, OHRQoL). The extent of the impairment is influenced by position and distribution of the affected or lost teeth [6]. Patients ask for solutions in order to compensate such kind of impairment and to re-establish their quality of life. Since the introduction of dental implants, one of the most common impairments—missing teeth—can be treated very effectively. Thus, the improvement in quality of life is a major treatment goal for implant-supported restorations and the patient satisfaction should be regarded as a central feature for treatment quality and success of therapy [7].

Patients’ self-awareness reflects their need for therapy; patient-reported outcome measures (PROMs) are able to show whether a treatment was able to improve their quality of life, for example by use of an implant-supported restoration. Additionally, PROMs can be used to evaluate the patients’ understanding of the performed treatment; a sufficiently good understanding can positively influence the commitment of the patient. Also, PROMs can be helpful for communication between decision-makers within the health care system, as therapies such as implant-supported restorations can be mediated in a tangible language that is easier to understand. In addition, PROMs are also potentially useful for the economic evaluation of various treatment methods [8].

Oral Health Impacted Profile (OHIP) is an internationally recognized instrument for evaluating the oral health-related quality of life [9–14]. For the purpose of an intercultural and international comparability of this psychometric instrument, this questionnaire was translated in 2002 with cultural adaptation into German. Four questions were added, which were specifically regarded as meaningful to the German population [15]. The culture-specific German version of the questionnaire has also proven its validity and sensitivity both in cross-sectional and longitudinal studies [16–18].

The Psychosocial Impact of Dental Aesthetics Questionnaire (PIDAQ) was developed in 2006 in order to investigate the effects of aesthetics on quality of life [19]. It is a well-validated psycho-social method for assessing the need for treatment as well as the treatment outcomes [19–23].

Since patients are no longer focused on the healing process with possible pain, swelling or bleeding, long-term follow-up studies over five years or more involving patient satisfaction survey provide reasonable information on real treatment benefit [4]. Although several long-term data regarding survival and success rates over periods of at least ten years are available for different implant systems [24–27], long-term results on patient satisfaction and individual improvement in quality of life are still rare [8]. Especially for the implant system XiVE and Frialite (Fa. Dentsply Sirona Implants, Mannheim, Germany), only one study concerning patient-reported outcomes is available with an observation period of 7.5 years [28].

Therefore, the aim of this study was to collect data of patient-reported outcomes in patients with XiVE and Frialite implants in a private periodontal practice 10 years after implant placement. Furthermore, factors potentially influencing patient satisfaction are investigated.

## Methods

The study was performed in accordance with the Declaration of Helsinki 1975, as revised in 2013, and was approved by the Institutional Review Board for Human Studies of the Medical Faculty of Heidelberg University (Application# S-210/2013). All patients were informed about possible risks and benefits as well as the procedures of the study and all gave written informed consent.

### Study population

As described in a previous paper on implant survival and success [29] all invited patients were treated in a private periodontal practice by implant placement of at least one XIVE or Frialite implant (Fa. Dentsply Sirona Implants, Mannheim, Germany). All patients were incorporated in an individual hygiene program prior to implant placement. If a periodontal disease was diagnosed, patients additionally received an active periodontal therapy (APT) prior to implant surgery. After implant placement all patients were invited in a recall program. 10 years ± 6 months after implant placement a re-examination was conducted.

All patients had to fulfill the following criteria:

- Available panoramic radiograph at implant placement (+ 3 months) and/or time of inserting the implant-supported prostheses (orthopantomogramm or x-ray)
- Available attachment level or panoramic radiograph/complete x-ray status to classify patient’s periodontal diagnosis at baseline
- Age ≥ 18 years at re-examination
- Non-pregnant or breastfeeding
- Partially edentulous dentition
- Completed questionnaires

### Clinical examination

After complete clinical re-examination performed by one independent examiner (AB) patients were asked to fulfill three questionnaires (clinical examination is described in detail in Bäumer et al. [29]):

#### Patients satisfaction

Patients answered six questions regarding their satisfaction on (1) general state, (2) phonetics, (3) chewing comfort, (4) stability, (5) cleanability, and (6) aesthetics using a four-grade categorizing scale: (a) “yes, very satisfied”, (b) “yes, mostly satisfied”, (c) “less satisfied”, (d) “not at all satisfied”.

In addition, patients were asked to mark for each question the respective Visual Analog Scale (VAS) [30] which is a 100 mm straight horizontal line with the left end indicating “not at all satisfied” and the right end “very satisfied”. The satisfaction value was determined by the distance from the left end of the scale to the mark in millimeters and expressed as percentage (10 mm corresponds to 10%, 20 mm 20%, etc.)

#### Oral health impact profile (OHIP)

Oral health-related quality of life (OHRQoL) was measured by using the German version of Oral Health Impact Profile (OHIP, 49 items) [13, 15]. For international comparability, the four German-specific questions were excluded. Patients were asked about the frequencies of complains during the last month.

A total of 49 OHRQoL-factors were rated on a scale of 0–4 (0 = “never”, 1 = “rarely”, 2 = “occasionally”, 3 = “often”, 4 = “very often”). There was no weighting of each single factor [15, 31]. The OHIP summary score was calculated as the sum of the 49 sub-scores (range 0–212) and characterized impairment. A higher OHIP score indicates a poorer OHRQoL. If more than five questions in total, two questions in a subgroup, or one of the three questions on problems specific to patients with prostheses were not answered, the patient was excluded. For all other cases, a statistical estimate gained via multiple imputation was used to supplement missing responses.

#### Psychosocial impact of dental aesthetics questionnaire (PIDAQ)

Influence of aesthetic perception of teeth in daily life was evaluated by consents to 23 statements. The consents to aesthetic-negative statements were rated on a scale of 0–4 (0 = “not at all”, 1 = “a little”, 2 = “somewhat”, 3 = “strongly” or 4 = “very strongly”). Since the subgroup “dental self-confidence” consisted of aesthetic-positive statements, the scales were rated reversed. Thus, a total score of 0 would represent an absolute satisfaction of aesthetics and a maximum total score of 115 would represent absolute dissatisfaction.

### Statistical analysis

Data was imported by two independent examiners (YW, DB) into the software “Microsoft® Excel for Mac 2011” (Microsoft Corporation, Redmond). For statistical analysis the statistics software R 3.2.2 (R Foundation for Statistical Computing, Vienna, Austria, www.r-project.com) was used. A descriptive analysis with mean, standard deviation, median, minimum, and maximum for continuous data was conducted. For categorial data percentages are given.

Statistical significance of potentially influencing factors (age, sex, smoking, peri-implantitis, implant position, type of restoration) on ordinal measurements of patient satisfaction was determined by ordinal or in one case logistic regression analysis. For continuous measurement variables (VAS, OHIP or PIDAQ) linear regression analyses were used. Estimates (odds ratio, mean difference) are given with corresponding 95% confidence interval and *p* value. If more than five OHIP score questions in total, two questions in an OHIP score subgroup, or one of the three questions on problems specific to patients with prostheses were not answered, the patient was excluded. For all other cases, a statistical estimate gained via multiple imputation was used to supplement missing responses. Thereby, the mice function in the mice package in R, which generates multivariate imputations by chained equations was used. Due to the descriptive nature of this study no adjustment for multiple testing was carried out and the significance level was set to be 0.05.

## Results

### Study population data

**Fig. 1:**
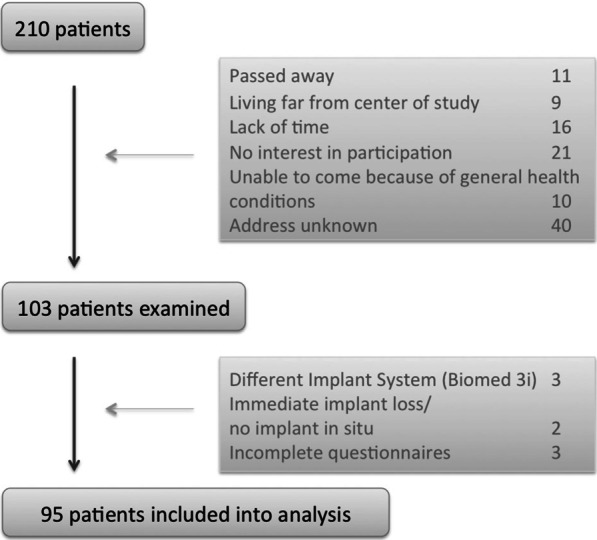
Recruitment of patient

103 out of 210 patients could be reexamined. In these analyses, 95 patients (60.0% female) are incorporated. Reasons for exclusion of eight patients are given in Fig. 1.

At baseline 33 patients showed a mild/moderate chronic periodontitis (ChP), 20 a severe ChP, 4 a localized aggressive periodontitis (AgP), and 11 a generalized AgP. All enrolled patients graduated at least from secondary school, 56.8% were also college/university graduates. At time of re-examination 9.5% of the patients were smokers and 44.2% were former smokers.

Patients were aged 28–86 years (mean 63.4 years, SD ± 10.4 years). Follow-up time was 10 years ± 6 month (mean 10.0 years, SD ± 0.3 years) after implant placement. The patients received one to nine implants (mean 2.5, SD ± 1.6). Only five patients (5.3%) got implant-supported prostheses, while 90 (94.7%) were restored with fixed single crowns or partial bridges. 40.0% of all patients suffered from mild peri-implantitis and 16.8% from moderate/severe peri-implantitis (Table 1).

**Table 1:** Patient characteristics

Patient characteristics	Total (*n* = 95)
Sex (female)	57 (60%)
Age (years)	63.4 ± 10.3 (range 28–86)
Follow-up time (years)	10 ± 0.3 (range 9.5–10.7)
Smoking at reexamination	
Current smoker	9 (9.5%)
Former smoker	42 (44.2%)
Never smoker	44 (46.3%)
Educational status	
Secondary school graduates	95 (100.0%)
College/university graduates	54 (56.8%)
Position of implants	
Anteriors	28 (29.5%)
Premolars	35 (36.8%)
Molars	32 (33.7%)
Prosthetic treatment	
Implant supported single crown	63 (66.3%)
Implant supported fixed prosthesis	27 (28.4%)
Removable denture	5 (5.3%)
Number of implants	2.5 ± 1.6 (range 1–9)
Implant type	
XiVE	84 (88.4%)
Frialit	11 (11.6%)
Peri-implantitis at reexamination	
No peri-implantitis	41 (43.2%)
Mild peri-implantitis	38 (40.0%)
Moderate/severe peri-implantitis	16 (16.8%)

^‡^Chronic periodontitis, ^§^aggressive periodontitis

### Patient satisfaction (PROMs)

**Fig. 2:**
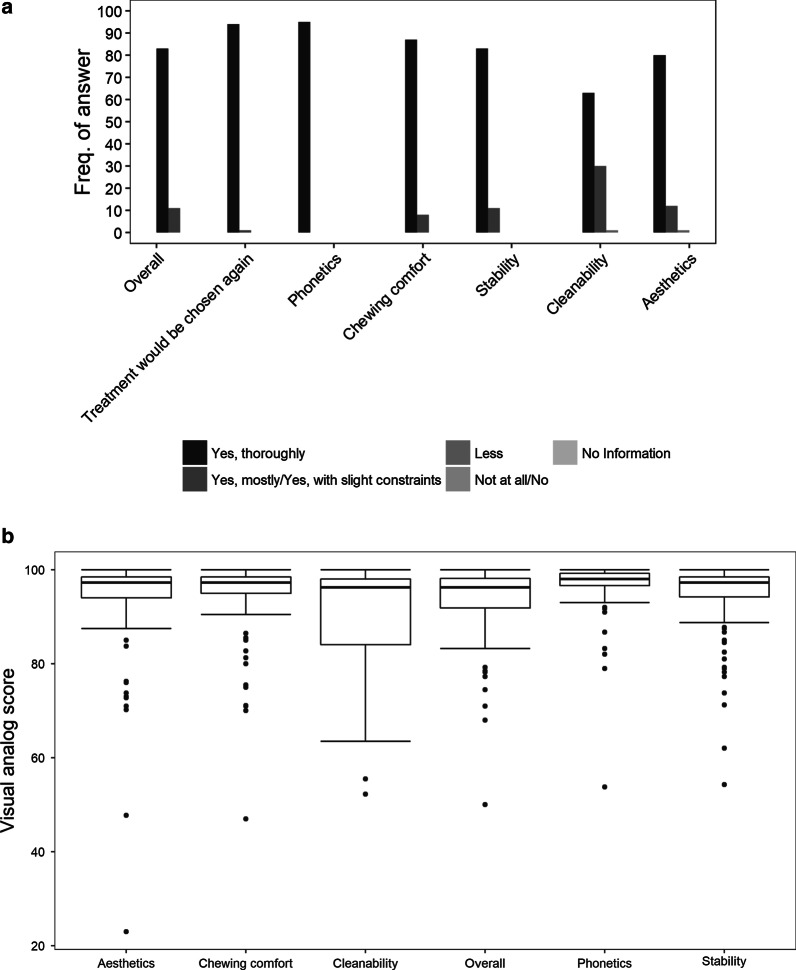
Patient satisfaction. **a** Bar plot depict the relative frequency of the ordinal answers possibility of patient satisfaction answered by ordinal categories. **b** Box and whisker plots show the distribution of the VAS of the patient satisfaction

- *Overall satisfaction.* 87.4% of all patients were strongly satisfied with their implant therapy. Only 11.6% responded with “yes, mostly” satisfied. Mean VAS was 93.1% (SD ± 9.4, median 96.3%, range 50.0–100%) (Fig. 2a, b and Additional file 1).
- *Phonetics.* 100% of the patients were strongly satisfied with their phonetics. Mean VAS was 96.7% (SD ± 5.8, median 98.0%, range 53.8–100%).
- *Chewing comfort*. 91.6% of patients were strongly satisfied with their chewing comfort, 8.4% of patients responded “yes, with slight restrictions”. Mean VAS was 94.2% (SD ± 8.6, median was 97.3%, range 47.0–100%).
- *Stability*. 87.4% of patients answered “yes, very”, 11.6% with “yes, for the most part”. Mean VAS was 94.1% (SD ± 8.3, median 97.3%, range 54.3–100%).
- *Cleanability*. 66.3% of the patients had no problems with the cleanability of their restoration. 31.6% of the patients had mostly no problems and one patient (1.1%) perceived the cleanability to be inferior. Mean VAS was 90.0% (SD ± 11.76, median 96.3%, range 52.3–100%).
- *Aesthetics*. 84.2% of the patients were very satisfied with the aesthetics of the implant-supported restoration, 12.6% responded to be mostly satisfied, and 1.1% of patients less satisfied with the aesthetics. Mean VAS was 93.3% (SD ± 11.37, median 97.0%, range 23.0–100.0%).

Patients who answered the questions with “very satisfied” did give higher VAS than patients that answer with “mostly satisfied” (Additional file 1: Fig. S1).

To summarize, these results indicate a profound patient satisfaction. This is also reflected by the fact that 98.9% of the patients would choose the treatment option of an implant-supported restoration again, if indicated. Only a single patient (1.1%) would not.

### OHIP

For four of the 95 patients, the OHIP scores could not be determined, because 5 or more questions of the questionnaire were not answered. The answers for all 49 questions of the 91 evaluable questionnaires are summarized in Table 2.

**Table 2:** Summary of the answers from 91 evaluable OHIP questionnaires

Prevalence of impairment in the past month	Never (%)	Rarely (%)	Occasionally (%)	Often (%)	Very often (%)
*Functional limitation*					
Difficulty chewing	67	24.2	8.8	0	0
Trouble pronouncing words	94.5	3.3	2.2	0	0
Noticed tooth that doesn't look right	89	3.3	6.6	1.1	0
Appearance affected	83.5	13.2	2.2	1.1	0
Breath stale	65.9	19.8	13.2	1.1	0
Taste worse	90.1	9.9	0	0	0
Food catching	28.6	23.1	30.8	12.1	5.5
Digestion worse	87.9	9.9	2.2	0	0
Dentures not fitting	98.9	0	1.1	0	0
*Physical pain*					
Painful aching	72.5	16.5	9.9	1.1	0
Sore jaw	70.3	17.6	11	1.1	0
Headaches	91.2	6.6	2.2	0	0
Sensitive teeth	59.3	16.5	20.9	3.3	0
Toothache	79.1	17.6	3.3	0	0
Painful gums	63.7	27.5	8.8	0	0
Uncomfortable to eat	85.7	6.6	5.5	2.2	0
Sore spots	67	16.5	16.5	0	0
Uncomfortable dentures	98.9	1.1	0	0	0
*Psychological discomfort*					
Worried by dental problems	68.1	13.2	15.4	2.2	1.1
Self-conscious	84.6	12.1	2.2	1.1	0
Dental problems made you miserable	86.8	13.2	0	0	0
Felt uncomfortable about the appearance	84.6	11	4.4	0	0
Felt tense	84.6	8.8	3.3	2.2	1.1
*Physical disability*					
Speech unclear	94.5	4.4	1.1	0	0
Others misunderstood	91.2	8.8	0	0	0
Less flavor in food	92.3	7.7	0	0	0
Unable to brush teeth	79.1	14.3	6.6	0	0
Avoid eating	90.1	5.5	2.2	2.2	0
Diet unsatisfactory	91.2	7.7	1.1	0	0
Unable to eat (dentures)	100	0	0	0	0
Avoid smiling	87.9	9.9	2.2	0	0
Interrupt meals	96.7	1.1	2.2	0	0
*Psychological disability*					
Sleep interrupted	87.9	7.7	4.4	0	0
Upset	90.1	6.6	3.3	0	0
Difficult to relax	63.7	27.5	8.8	0	0
Depressed	87.9	8.8	3.3	0	0
Concentration affected	87.9	11	1.1	0	0
Been embarrassed	91.2	7.7	1.1	0	0
*Social disability*					
Avoid going out	97.8	2.2	0	0	0
Less tolerant of others	84.6	14.3	1.1	0	0
Trouble getting on with others	89	11	0	0	0
Irritable with others	86.8	11	2.2	0	0
Difficulty doing jobs	89	11	0	0	0
*Handicap*					
Your general health has worsened	85.7	9.9	4.4	0	0
Financial loss	72.5	18.7	8.8	0	0
Unable to enjoy people’s company	89	8.8	0	1.1	1.1
Life unsatisfying	76.9	19.8	3.3	0	0
Unable to function	89	11	0	0	0
Unable to work	91.2	8.8	0	0	0

Mean OHIP score was 11.3 (SD ± 10.8, median 9, range 0–45, 1st quantil 3, 3rd quantil 16.5). The median of 9.0 signifies that half of all patients rank on the top 5% of the OHIP scale. 11 patients (11.6%) experienced no impairment of OHRQoL in the last month, thus exhibit the best possible OHIP score of zero. In sum, this indicates once more that therapy with implant-supported restorations is a favorable treatment option for eligible patients.

The most common limitations were reported in the subgroups “*functional limitations*” and “*physical pain*”. Almost half of all patients (48.4%) complained about “food catching between teeth or underneath denture” (functional limitations) “occasionally”, “often” or “very often”. Prevalence between 15.0% and 25.0% was shown by impairments “bad breath” (functional limitations), “sensitive teeth” (physical pain), “sore spots” (physical pain) and “worried by problems” (psychological discomfort).

### Psychosocial impact of dental aesthetics questionnaire: PIDAQ

The average PIDAQ score of all 95 patients was 20.5 (SD ± 11.3, median 17.0, range 0–52). One patient was perfectly satisfied with the aesthetics and showed a score of 0. The frequencies of the answer categories to all 23 questions are summarized in Table 3.

**Fig. 3:**
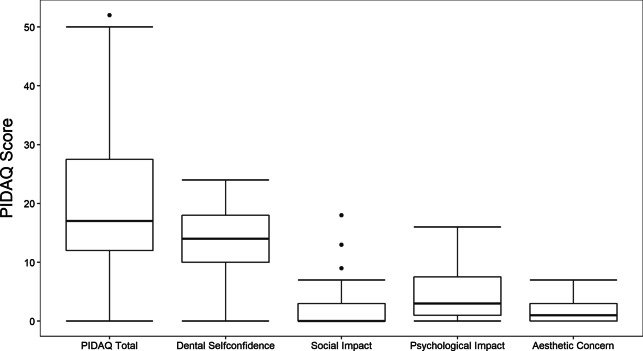
PIDAQ. Box and whisker plots show the distribution of the PIDAQ score and its subgroups

- *Dental self-confidence*. Mean score was 13.9 (SD ± 5.2, median 14.0, range 0–24). The statements of this subgroup, which describe a positive dental self-awareness, do not have many agreements as presented in Table 3.
- *Psychological impact.* Mean score was 4.6 (SD ± 4.4, median 3.0, range 0–16). In this subgroup impairments have a relatively high prevalence.
- *Social impact.* Mean score was 1.8 (SD ± 2.9, median 0.0, range 0–18).
- *Aesthetic concern.* Mean score was 1.7 (SD ± 1.9, median 1.0, range 0–7). This indicates that patients were less affected from social impact and aesthetic concern. Most negative statements in these two subgroups did not find more than 5% consents for “strongly” or “very strongly” (Fig. 3).

**Table 3:** Summary of distribution to answers to all 23 PIDAQ-Statements

Statements	Very stronly (%)	Strongly (%)	Somewhat (%)	A little (%)	Not at all (%)
*Dental self-confidence*					
Proud of own teeth	5	14	30	17	33
Like to show own teeth	6	31	28	16	18
Pleased to see own teeth in mirror	7	21	33	26	13
Own teeth look attractive to others	3	5	35	23	35
Satisfied with own teeth’s appearance	13	39	19	19	10
Find own teeth position nice	8	23	30	19	21
*Social impact*					
Hold back while smile	0	1	6	9	84
Concerned what others think about my teeth	0	1	6	7	85
afraid of offensive remarks from others	0	1	1	3	95
Inhibited in social contacts because of own teeth	1	0	0	8	91
Hiding own teeth with hand	0	0	0	5	95
People stare at my teeth	0	2	3	8	87
Irritated on remarks	2	3	13	17	64
other gender find my teeth ugly	0	0	4	7	89
*Psychological impact*					
Envy others for their teeth	5	13	21	19	41
Distressed because of others’ nice teeth	1	9	16	17	57
Unhappy about own teeth's appearance	0	1	10	27	63
Think others have nicer teeth	1	4	20	16	59
Feel bad about own teeth's appearance	0	1	7	24	68
Wish own teeth to look better	4	4	18	32	42
*Aesthetic concern*					
Don’t like own teeth in mirror	0	3	12	26	59
Don’t like own teeth on photos	0	3	15	16	65
Don’t like own teeth on video	1	2	8	24	65

### Influencing factors on PROMs

*Patient satisfacti*o*n*. A slight tendency that presence of a moderate/severe peri-implantitis had an impact on satisfaction could be detected (Table 4). Patients with moderate/severe peri-implantitis showed a lower overall satisfaction compared to patients with no peri-implantitis on the ordinal scale (ordinal: *p* = 0.012, VAS: *p* = 0.026). A higher odds ratio refers to a higher ordinal score that corresponds to a lower satisfaction whereas a lower VAS refers to a lower satisfaction. For the items “less stability” and “less satisfaction with aesthetics” this tendency is also seen for both the ordinal scale and VAS (*p* < 0.001, VAS: *p* = 0.010; *p* = 0.050, VAS: *p* = 0.009; respectively). For the small subgroup of patients with removable prostheses, a statistical significant effect was also observed for the item ‘less stability’ on the ordinal scale (*p* = 0.034). Younger patients showed rather ‘less satisfaction with aesthetics’ on the VAS (*p* = 0.037). Regarding the item ‘phonetic’ patients with implants in the frontal region were less satisfied on VAS (*p* = 0.017).

**Table 4:** Multiple regression analyses regarding factors influencing patient satisfaction (a, b), OHRQoL and psychological impact of aesthetic appearance (c)

	Overall				Chewing comfort				Stability				Cleanability				Aesthetics			
	OR	LL	UL	*p* value	OR	LL	UL	*p* value	OR	LL	UL	*p* value	OR	LL	UL	*p* value	OR	LL	UL	*p* value
*(a) Ordinal*																				
Sex (male vs. female)	0.543	0.148	2.962	0.388	0.653	0.132	3.213	0.59	0.444	0.137	3.966	0.312	1.152	0.096	2.757	0.758	0.673	0.031	2.899	0.509
Age	1.007	0.066	6.225	0.839	0.973	0.904	1.044	0.438	0.98	0.029	5.954	0.554	1.014	0.547	4.145	0.522	0.975	0.381	6.466	0.396
*Smoker*																				
Former smoker versus never smoker	0.693	0.314	10.08	0.624	0.631	0.079	3.842	0.626	0.78	0.543	31.3	0.767	0.962	0.314	3.965	0.936	1.384	1.027	20.85	0.609
Current Smoker versus Never Smoker	0.826	1.721	55.56	0.865	1.192	0.056	10.05	0.884	0.538	5.317	428.6	0.645	0.587	0.361	3.664	0.523	0.344	0.462	9.262	0.351
*Peri-implantitis*																				
Mild versus No	1.714	0.336	20.42	0.5291	1.279	0.194	8.469	0.792	3.566	0.880	61.81	0.199	1.494	0.369	4.539	0.434	1.544	0.136	4.699	0.540
Moderate/Servere versus No	8.896	0.405	23.65	0.0115	1.055	0.048	9.990	0.965	37.22	0.111	9.173	0.001	1.149	0.291	2.832	0.828	4.506	0.339	5.631	0.047
*Position of implant*																				
Premolar versus Anterior	2.282	0.095	3.924	0.413	1.478	0.169	16.48	0.729	6.170	0.008	0.694	0.084	1.137	0.035	2.513	0.827	1.974	0.026	5.812	0.367
Molar versus Anterior	2.613	0.006	4.102	0.337	0.595	0.058	5.659	0.642	0.96	0.003	3.817	0.969	1.276	0.471	2.885	0.701	0.806	0.202	2.199	0.809
*Prosthetic treatment*																				
Bridge versus Single Crown	0.647	0.128	2.181	0.637	0.331	0.016	2.724	0.354	0.098	0.083	2.088	0.034	0.914	0.972	1.06	0.875	1.365	0.919	1.034	0.660
Removable denture versus Single Crown	0.218	0.946	1.074	0.340	1.549	0.063	19.37	0.744	0.151	0.916	1.048	0.309	0.374	0.369	2.504	0.353	0.466	0.398	4.987	0.587

	Overall				Phonetics				Chewing comfort				Stability				Cleanablity				Aesthetics			
	Estimate	LL	UL	*p* value	Estimate	LL	UL	*p* value	Estimate	LL	UL	*p* value	Estimate	LL	UL	*p* value	Estimate	LL	UL	*p* value	Estimate	LL	UL	*p* value
*(b) Visual analoge scale*																								
Sex (male vs. female)	0.221	− 3.709	4.152	0.911	1.334	− 1.079	3.747	0.275	− 0.001	− 3.717	3.715	1.000	1.799	− 1.585	5.182	0.294	− 0.858	− 6.156	4.44	0.748	− 1.076	− 5.775	3.624	0.650
Age	0.057	− 0.134	0.247	0.556	− 0.015	− 0.132	0.102	0.800	− 0.001	− 0.181	0.179	0.993	0.021	− 0.1423	0.185	0.801	− 0.060	− 0.319	0.198	0.644	0.241	0.015	0.467	0.037
*Smoker*																								
Former smoker versus never smoker	− 2.896	− 7.138	1.345	0.178	1.366	− 1.238	3.969	0.300	1.122	− 2.888	5.132	0.58	− 2.771	− 6.434	0.892	0.136	1.326	− 4.484	7.136	0.651	− 0.211	− 5.312	4.89	0.935
Current smoker versus never smoker	3.051	− 3.688	9.791	0.371	1.255	− 2.881	5.392	0.548	1.014	− 5.358	7.385	0.753	− 3.702	− 9.486	2.082	0.207	4.615	− 4.478	13.71	0.316	5.498	− 2.516	13.51	0.176
*Peri-implantitis*																								
Mild versus no	− 3.146	− 7.566	1.275	0.161	− 0.37	− 3.083	2.345	0.788	0.41	− 3.769	4.59	0.846	− 3.459	− 7.271	0.354	0.075	− 1.931	− 7.876	4.015	0.520	− 1.484	− 6.83	3.862	0.582
Moderate/servere versus no	− 6.262	− 11.75	− 0.7723	0.026	− 2.426	− 5.796	0.944	0.156	− 4.616	− 9.806	0.575	0.081	− 6.253	− 10.96	− 1.541	0.010	− 1.895	− 9.456	5.667	0.620	− 8.859	− 15.41	− 2.313	0.009
*Position of implant*																								
Premolar versus anterior	− 1.9	− 7.018	3.218	0.463	2.629	− 0.513	5.770	0.100	2.045	− 2.794	6.884	0.403	− 3.425	− 7.836	0.985	0.126	− 0.060	− 6.965	6.844	0.986	− 0.681	− 6.886	5.524	0.828
Molar versus anterior	− 0.152	− 5.635	5.33	0.956	4.129	0.764	7.495	0.017	2.429	− 2.754	7.612	0.354	2.692	− 2.016	7.399	0.259	− 0.453	− 7.832	6.925	0.903	2.3	− 4.373	8.974	0.495
*Prosthetic treatment*																								
Bridge versus single crown	1.568	− 3.426	6.562	0.534	2.730	− 0.335	5.796	0.080	0.410	− 4.312	5.131	0.864	4.235	− 0.080	8.55	0.054	3.047	− 3.728	9.821	0.374	1.323	− 4.641	7.288	0.660
Removable denture versus single crown	3.986	− 3.962	11.93	0.322	2.905	− 1.974	7.783	0.240	1.441	− 6.073	8.956	0.704	1.589	− 5.232	8.41	0.6444	1.654	− 9.033	12.34	0.759	2.498	− 6.991	11.99	0.602

	OHIP				PIDAQ			
	Estimate	LL	UL	*p* value	Estimate	LL	UL	*p* value
*(c) OHIP and PIDAQ*								
Sex (male vs. female)	2.846	− 2.172	7.865	0.263	1.535	− 3.283	6.352	0.528
Age	0.082	− 0.161	0.324	0.506	0.029	− 0.204	0.263	0.802
Smoker former smoker versus never smoker	1.546	− 3.869	6.962	0.572	− 0.771	− 5.97	4.428	0.769
Current smoker versus never smoker	1.509	− 7.096	10.11	0.728	2.099	− 6.161	10.36	0.615
Peri-implantitis mild versus no	− 0.445	− 6.089	5.2	0.876	− 2.833	− 8.252	2.586	0.302
Moderate/servere versus no	5.01	− 1.999	12.02	0.159	2.256	− 4.473	8.985	0.507
Position of implanat premolar versus anterior	− 1.334	− 7.869	5.201	0.686	− 4.012	− 10.28	2.262	0.207
Molar versus anterior	0.174	− 6.825	7.174	0.961	− 1.186	− 7.905	5.534	0.727
Prosthetic treatment Bridge versus Single Crown	0.427	− 5.949	6.803	0.894	− 0.440	− 6.561	5.681	0.887
Removable denture versus single crown	2.466	− 7.682	12.61	0.630	− 13.57	− 23.31	− 3.826	0.007

*LL* lower limit of 95% confidence interval, *UL* upper limit of 95% confidence interval

Two models, i.e. the model for “Treatment would be chosen again” and ordinal “Phonetics”, could not be conducted because of the too negligible differences between patient choices.

*OHIP* and *PIDAQ*. Statistical analysis showed no statistically significant impact on OHIP. On *PIDAQ* the factor removable prostheses had an impact (*p* = 0.007).

## Discussion

At the VIII. European Workshop on Periodontology Tonetti and Palmer [5] proposed that clinical research in implant dentistry should not only assess biological complications (like implant loss, peri-implant mucositis or peri-implantitis) and technical complications, but also focus on patient’s satisfaction and aesthetic outcomes. Therefore, next to biological and technical complications contained in a previous paper (Bäumer et al. [29]), long-term study data on patient satisfaction with implant-supported restorations 10 years after implant placement are presented.

### Patient satisfaction

Overall, the patient survey shows a very high degree of satisfaction with implant-supported restorations, both functionally and aesthetically. All patients were highly satisfied with their phonetics and most with their chewing comfort, cleanability and stability of the restoration. Except for one patient, all were very or mostly satisfied with the aesthetics and would choose this treatment again. These results are comparable with a cohort study from Switzerland [32] with 104 patients recruited 5–15 years (mean: 10.2 years) after implantation for follow-up examination. In both studies, over 90% of patients are fully satisfied with implant-supported restorations. This is also in accordance with another retrospective study from France, which reported on satisfaction 10–16 years after implantation [24]. Sligthly lower subjective satisfaction of patients were found in a retrospective study from Germany with 37 hypodontia patients recruited 0.5–16 years after implantation, however the results were then improved by using objective assessments [33]. Despite of the small sample size of patients with implant-supported removable prostheses in our study (only five patients), a great OHRQoL among the patients with implant-supported removable prostheses could also be confirmed, even though none of the removable prostheses were free from clinical complications and require more prosthetic maintenance [34].

In OHIP some impairments could be found in this patient group. For example, half of the patients complained about food impaction between their teeth/implants. Additionally, to the fact that interdental spaces between implants and teeth—especially in the molar regions—are wider than in natural dentitions, most of the patients were periodontally compromised, which include wider interdental spaces with increased food catching problems also between teeth. Further impairments such as sensitive teeth are also caused by recessions in periodontally compromised situations and do not have an association with the conducted implant therapy.

The similarity of the results regarding patient-reported quality of life (OHIP) in this study with the result found for the population with natural teeth without dentures from the German cross-sectional study also reflects the positive effect of implant-supported restorations for patients [17]. Based on frequency distribution of the OHIP scores, the patient population presented here compares better to the group with natural teeth (without removable dentures) than to the group with natural teeth and removable dentures. The average age of the patients in this study is higher than the patient group studied by John et al. [17]. Also, the education level of the patients is higher in comparison. Only 8.1% of all subjects in the German cross-section study obtained a college or university degree, whereas more than 57% of patients in this study graduated from college or university. The gender distribution was slightly different (60% women in our study, 52% women by John et al.).

Thus, it can be assumed that by means of fixed implant-supported restorations, patients are almost as satisfied as the population with natural teeth regarding function and aesthetics. This is in agreement with two other studies using OHIP-G14, a short-form of OHIP in German with only 14 questions. They report an improved quality of life for patients that received therapies with implant-supported restorations. One of the studies started out from a periodontally compromised situation [16]. The other one surveyed the identical implant system (XiVE), but with an observation period of 7.5 years [28]. Compared to the OHIP-Scores (32.6 ± 30.1) reported in a 10-year follow-up study in the Netherlands on 28 oligodontia patients rehabilitated with implant-based fixed prosthodontics [35], the results of the present study (10.8 ± 10.8) appear more homogenous and patients reported higher satisfaction. Aesthetic satisfaction seems to be more challenging with oligodontia patients, since bone augmentation is almost mandatory due to the lack of native bone, both in vertical and horizontal dimension. Nevertheless, both studies suggested implant treatment to be a predictable and satisfactory treatment modality for missing teeth.

PIDAQ shows implant-supported restorations positively affect not only oral and dental health, but also the dental aesthetics-related quality of life. Only *“*dental self-confidence*”* showed reduced results, and reflects the self-critical attitude of the patient population. Developed for the assessment of psychosocial impact of dental aesthetics after orthodontic treatments, this questionnaire could only be found once in the literature relating to implantation: gender and educational differences were suggested to exist [21]. Gender influence has not been confirmed in the present study, but a direct comparison between these two studies is difficult, since Chen et al. [21] weighted the single questions additionally.

All together, this indicates again that therapy with implant-supported restorations is a favorable treatment option for eligible patients.

### Limitations

The cross-sectional nature of data collection in this study as well as the absence of baseline data to assess changes due to the treatment ‘implant placement’ is a major limitation in this study. The patient expectation at baseline regarding the therapeutic outcome, which may also influence satisfaction [36], is not available. To overcome these limitations and also to avoid a possible ‘recall bias’ i.e. to recruit more satisfied than dissatisfied patients, since dissatisfied patients might seek further treatment somewhere else [37], prospective studies are required in the future.

Also, the examined patients did not represent an average population. All patients were highly educated, showed a high average age (63.4 ± 10.4 years) and most patients were restored with fixed-implant supported restorations.

Furthermore, the analysis regarding influencing factors on patient’s satisfaction has to be interpreted with care. On the one hand, there might be relevant factors, that were excluded in multiple regression analyses, and other not relevant factors were included. On the other hand, included factors were biased due to study design. So, in case of the factor peri-implanitits in this study it has to be borne in mind that patients were informed on their peri-implantitis before they completed the questionnaires. Accordingly, an association should be assumed.

## Conclusions

Patients treated with XIVE and Frialite implants showed a high patient’s satisfaction 10 years after implant placement. The factors moderate/severe peri-implantitis, age, implant position and type of restoration were identified as potentially influencing patient’s satisfaction, but these results have to be interpreted with care due to the cross-sectional design.

## Supplementary Information

**Additional file 1.** Patients satisfaction: Comparison of answers regarding PROMs given on questionnaire and VAS.

## Data Availability

The datasets used and analysed during the current study are available from the corresponding author on reasonable request.

## Abbreviations

AG: Aggressive periodontitis
APT: Active periodontal therapy
CI: Confidence interval
CP: Chronic periodontitis
Fa.: Firm
OHIP: Oral health impact profile
OHRQoL: Oral health-related quality of life
PIDAQ: Psychosocial impact of dental aesthetics questionnaire
PROM: Patient-reported Outcome Measures
SD: Standard deviation
VAS: Visual analog scale
