# Supplementary figures and images for: Patient satisfaction and oral health-related quality of life 10 years after implant placement
Source: BMC Oral Health. 2021 Jan 14;21:30. doi: 10.1186/s12903-020-01381-3 (PMC7807859; doi:10.1186/s12903-020-01381-3)

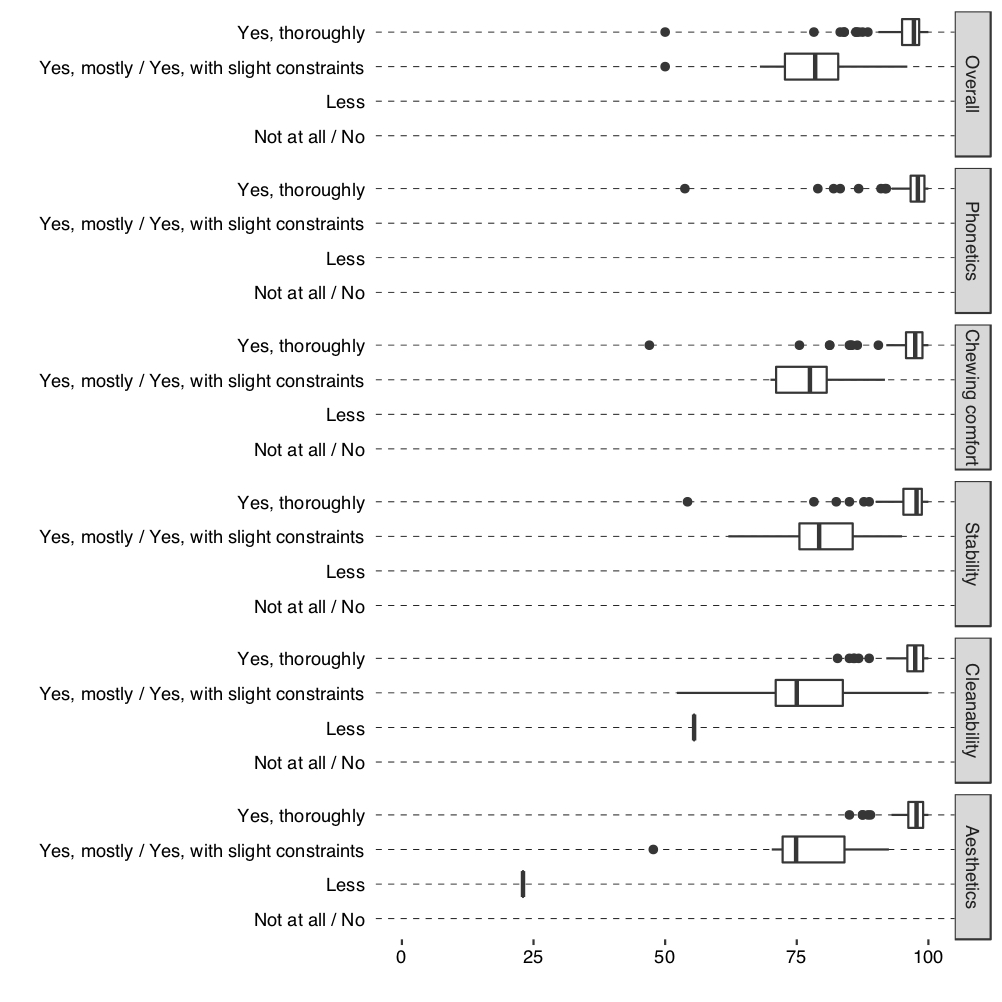

Supplement: Supplementary file 1 — Additional file 1. Patients satisfaction: Comparison of answers regarding PROMs given on questionnaire and VAS. [file 12903_2020_1381_MOESM1_ESM.jpg]
